# Protocol for real-time imaging of membrane fission by mitofissin

**DOI:** 10.1016/j.xpro.2023.102590

**Published:** 2023-09-21

**Authors:** Tatsuro Maruyama, Nobuo N. Noda.

**Affiliations:** 1 Institute of Microbial Chemistry (BIKAKEN), Shinagawa-ku, Tokyo 141-0021, Japan; 2 Institute for Genetic Medicine, Hokkaido University, Sapporo, Hokkaido 060-0815, Japan

**Keywords:** Biophysics, Microscopy, Protein Biochemistry

## Abstract

Yeast mitofissin Atg44 is a mitochondrial intermembrane space protein that causes membrane fission required for mitophagy. Here, we present a protocol for observing Atg44-mediated membrane fission. We describe steps for recombinant Atg44 purification, lipid nanotube preparation as model membranes, and Atg44-mediated membrane fission real-time observation. We then detail procedures for tube radius estimation using confocal microscopy. This protocol can also be adapted to the study of membrane fission by other proteins.

For complete details on the use and execution of this protocol, please refer to Fukuda et al. (2023).^1^

## Highlights

• Purification of recombinant mitofissin
• Observation of mitofissin-mediated membrane fission using lipid nanotubes
• Tube radius estimation from fluorescence intensity

## Abstract (editor highlights)

Publisher’s note: Undertaking any experimental protocol requires adherence to local institutional guidelines for laboratory safety and ethics.

## Graphical abstract

**Figure:**
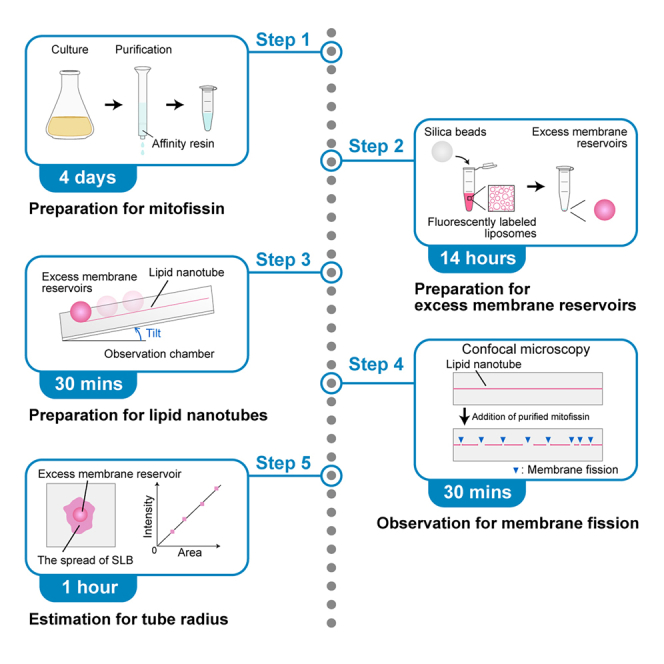

## Before you begin

The protocol below provides a comprehensive guide to conducting *in vitro* membrane fission observation using Atg44 as an example. This protocol uses lipid nanotubes as model membranes. The nanotubes can be prepared by the interaction between lipids and glass surface without requiring special or expensive devices. The lipid composition of the nanotubes is intentionally designed to mimic that of the inner mitochondrial membrane, ensuring relevance to the intermembrane space where Atg44 is located. However, the lipid composition can be adjusted to suit the research interests. Notably, we have successfully generated lipid nanotubes using various lipid compositions such as 1:1 of phosphatidylcholine (PC) and phosphatidylethanolamine (PE), 2:1:1 of PC, PE and an acidic phospholipid like phosphatidylinositol (PI), implying the flexibility of this protocol. Moreover, this protocol can be modified and applied to assess the fission activity of other proteins. Sample preparation, appropriate lipid composition selection, and necessary cofactor identification relevant to membrane fission observation mediated by the protein of interest should be carefully considered before starting the protocol. Appropriate starting lipid composition can be studied using a liposome flotation assay that reveals the binding of the protein of your interest to the lipid membranes.

### Purification of recombinant mitofissin, SpAtg44

**Timing: ∼4 days**

Recombinant Atg44 from *Schizosaccharomyces pombe* (hereafter, denoted as SpAtg44) is overexpressed in *Escherichia coli* (*E. coli*) with a maltose-binding protein (MBP) fused to its N terminus. PreScission protease recognition sequence is inserted between MBP and SpAtg44 so that MBP can be removed by the protease treatment. PreScission protease is a glutathione-S-transferase-tagged human rhinovirus 3C protease that is also commercially available. SpAtg44 is purified to be homogeneous as shown in size exclusion chromatography.^1^

1. Purify recombinant SpAtg44.
  a. Transform competent *E. coli* C41(DE3) cells with a plasmid, in-house modified pET15b-SpAtg44.
  b. Inoculate the transformant cells into 10 mL of Luria broth (LB) medium supplemented with 100 mg/L of ampicillin in a 50-mL plastic tube and grow them at 37°C for ∼16 h.
    ***Note:*** Select transformants in an ampicillin-containing medium whose resistance is encoded in the plasmid.
  c. Transfer the culture into 1 L of LB medium supplemented with 100 mg/L of ampicillin in a 2-L baffled flask.
  d. Grow the cells at 37°C until the optical density at 600 nm of the culture reaches ∼0.8.
  e. Induce the protein expression by adding isopropyl β-D-thiogalactopyranoside (IPTG) from 0.5 M of stock to a final concentration of 0.5 mM, followed by the culture at 20°C for ∼20 h.
  f. Harvest the cells by centrifugation at 4,000 × g for 10 min at 4°C.
  g. Decant the supernatant and resuspend the cell pellet in 35 mL of phosphate buffer.
    ***Note:*** The cell suspension can be stored at −80°C.
    ***Note:*** Purification procedures are recommended to be conducted in chilled conditions, such as on ice or in a cold room.
  h. Supplement the cell suspension with 1.0 mM of phenylmethylsulfonyl fluoride (PMSF) from 0.1 M of stock in ethanol.
  i. Lyse the cells using a tip sonicator (500 W, 20 kHz) with the tube immersed in ice water. Sonication is performed with 50% amplitude in 1-s bursts followed by 4-s rests for 15 min.
    ***Note:*** Ultrasonic sound with high power can be harmful for hearing.
  j. Centrifuge the lysate at 38,000 × g for 30 min at 4°C.
  k. Load the supernatant on ∼3 mL of amylose resin pre-equilibrated with phosphate buffer in a 20-mL gravity column without disturbing the resin.
  l. Wash the resin with 40 mL of phosphate buffer.
    **CRITICAL:** High salt concentration (>1 M of NaCl) in the wash step is essential to homogeneously purify SpAtg44.
  m. Elute the bound proteins by adding 10 mL of phosphate buffer supplemented with 10 mM of maltose.
  n. Supplement the eluate with homemade PreScission protease (also commercially available from Cytiva) at a final concentration of ∼1 μM, followed by incubation at 4°C for ∼16 h.
  o. Separate the sample on SDS-PAGE with 15% polyacrylamide gel. Stain with Coomassie blue G-250 to check MBP cleavage.
  p. Load the sample on ∼3 mL of Ni-NTA resin pre-equilibrated with phosphate buffer in a 20-mL gravity column without disturbing the resin.
    ***Note:*** SpAtg44 can bind to Ni-NTA resin without His-tag since the residues are structurally clustered in the octameric architecture.
  q. Wash the resin with 30 mL of 4-(2-hydroxyethyl)-1-piperazineethanesulfonic acid (HEPES) buffer supplemented with 10 mM of imidazole.
  r. Elute the bound protein by adding 10 mL of HEPES buffer supplemented with 200 mM imidazole.
  s. Separate the sample on SDS-PAGE. Stain with Coomassie blue G-250 to check the purity.
  t. Concentrate the elution to ∼250 μL using a 10-kDa centrifugal device at 4°C.
  u. Add 10 mL of HEPES buffer to the concentrated sample and concentrate it again.
  v. Repeat steps 1t and 1u three times to exchange the buffer into the HEPES buffer.
  w. Divide the purified SpAtg44 into small aliquots and freeze at −80°C ∼10 mg/L culture of SpAtg44 can be obtained.
    ***Note:*** Purification by size exclusion chromatography is optional.
    ***Note:*** Avoid repeating freeze-thaw cycles of purified SpAtg44.

### Preparation for liposomes

T**iming: ∼14 h**

Liposomes are used for lipid nanotube preparation using excess membrane reservoirs as described below. The fluorescent-labeled phospholipid is included in the lipid composition to observe lipid nanotubes under a confocal laser scanning microscope. The lipid composition can be changed according to the experimental plan.

2. Prepare liposomes.
  a. Prepare lipid mixture solution for excess membrane reservoirs in a 5-mL glass vial as shown below.
    ***Note:*** It is recommended to dispense lipid solution using glass syringes.
    **CRITICAL:** Chloroform can harm the eyes, skin, liver, kidneys, and nervous system and can be toxic if inhaled or swallowed, thus should be disposed of following local regulations.
  b. Dry chloroform by gently applying nitrogen gas through the Pasteur pipette while rotating the glass vial to create thin lipid films.
    **CRITICAL:** This step should be conducted in a fume hood.
  c. Put the glass vial in a desiccator connected to a rotary pump for >12 h to completely remove chloroform.
  d. Add 400 μL of water to the lipid films containing 100 nmol of total lipids in the vial.
  e. Agitate lipid films using a vortex mixer to produce a suspension.
  f. Transfer the suspension to a 1.5-mL plastic tube.
  g. Sonicate the suspension using a handheld tip sonicator until transparent. Sonication is performed with ∼40% output power in 10-s bursts followed by 10-s rests.
    ***Note:*** Ultrasonic sound with high power can be harmful for hearing.
  h. Centrifuge the liposome solution at 18,000 × g for 15 min at 25°C to remove debris.
  i. Transfer the supernatant containing liposomes to a fresh 1.5-mL plastic tube.
    ***Note:*** Liposome solution should be prepared just before use.

## Key resources table

**Table:** 

REAGENT or RESOURCE	SOURCE	IDENTIFIER
**Bacterial and virus strains**		

*E. coli* C41(DE3)	Sigma-Aldrich	Cat# CMC0017

**Chemicals, peptides, and recombinant proteins**		

Tryptone	Nacalai Tesque	Cat# 35640-95
Yeast extract	Nacalai Tesque	Cat# 15838-45
NaCl	Nacalai Tesque	Cat# 31319-45
Ampicillin	Nacalai Tesque	Cat# 19769-22
IPTG (isopropyl β-D-thiogalactopyranoside)	Nacalai Tesque	Cat# 19742-07
PMSF (phenylmethylsulfonyl fluoride)	Nacalai Tesque	Cat# 27327-52
HEPES	Nacalai Tesque	Cat# 17546-05
Na_2_HPO_4_·12H_2_O	Nacalai Tesque	Cat# 31722-45
KH_2_PO_4_	Nacalai Tesque	Cat# 28720-65
KCl	Nacalai Tesque	Cat# 28513-85
NaOH (sodium hydroxide)	Nacalai Tesque	Cat# 31511-05
Maltose	Nacalai Tesque	Cat# 21117-82
Imidazole	Nacalai Tesque	Cat# 08787-35
HCl (hydrochloric acid)	Nacalai Tesque	Cat# 18402-45
Amylose resin	New England Biolabs	Cat# E8021
Ni-NTA superflow	QIAGEN	Cat# 30410
Chloroform	Nacalai Tesque	Cat# 08415-95
1-palmitoyl-2-oleoyl-sn-glycero-3-phosphocholine (POPC)	Avanti Polar Lipids	Cat# 850457
1-palmitoyl-2-oleoyl-sn-glycero-3-phosphoethanolamine (POPE)	Avanti Polar Lipids	Cat# 850757
L-α-phosphatidylinositol (PI)	Avanti Polar Lipids	Cat# 840042
1-palmitoyl-2-oleoyl-sn-glycero-3-phospho-L-serine (POPS)	Avanti Polar Lipids	Cat# 840034
1-palmitoyl-2-oleoyl-sn-glycero-3-phosphate (POPA)	Avanti Polar Lipids	Cat# 840857
1′,3′-bis[1,2-dioleoyl-sn-glycero-3-phospho]-glycerol (CL)	Avanti Polar Lipids	Cat# 710335
1,2-dioleoyl-sn-glycero-3-phosphoethanolamine-N-(lissamine rhodamine B sulfonyl) (liss Rhod PE)	Avanti Polar Lipids	Cat# 810150
Silica beads (50 μm)	Nanocs	Cat# Si01-50u-1
BSA	Wako	Cat# 016-15091
PreScission protease	Cytiva	Cat# 27-0843-01

**Recombinant DNA**		

pET15b-SpAtg44	Fukuda et al.^1^	N/A

**Software and algorithms**		

Fiji	Schindelin et al.^2^	http://fiji.sc/Fiji
ImageJ	National Institutes of Health	https://imagej.nih.gov/ij
FV31S-SW	Evident	https://www.olympus-lifescience.com/
Excel	Microsoft	https://www.microsoft.com/

**Other**		

Milli-Q water	Merck Millipore	N/A
2 L baffled flask	Sibata	Cat# 016310-2000
20 mL gravity column	Bio-Rad	Cat# 7321010
Amicon Ultra-15 10 kDa centrifugal device	Merck Millipore	Cat# UFC901024
Desiccator	Merck	Cat# BAF424002141
Rotary pump	Phil	Cat# P100D
High-speed centrifuge	Beckman Coulter	N/A
Super-speed centrifuge	Thermo Fisher Scientific	Cat# 75006590
Sonicator	Qsonica	Cat# Q500
Handheld sonicator	SMT	Cat# UH-50
Vortex mixer	M&S Instruments	Cat# 0K-0500-902
5 mL glass vial	As One	Cat# 9-851-04
Microscope cover glass (24 × 24 mm)	Matsunami Glass	Cat# C024241
Microscope slide glass	Matsunami Glass	Cat# S011110
Silicone rubber sheet	As One	Cat# 6-611-02
Glass capillary	Narishige	Cat# G-100
Puller	Narishige	Cat# PC-100
Capillary cleaving tool	Supelco	Cat# 23740-U
Dry vacuum pump	Ulvac	N/A
Differential pressure transducer	Validyne	N/A
Pressure amplifier	Krone	N/A
Digital multimeter	Custom	N/A
Confocal laser scanning microscope, FV3000RS	Evident	N/A

## Materials and equipment

**Table:** LB medium

Reagent	Amount
Tryptone	10 g
Yeast extract	5 g
NaCl	5 g
Water	Add 1 L
**Total**	**1 L**

Autoclave and cool down to 20°C–25°C before use. Do not store the medium in a baffled flask.

**Table:** PBS buffer (×10)

Reagent	Amount
Na_2_HPO_4_ 12H_2_O	29 g
KH_2_PO_4_	2 g
NaCl	80 g
KCl	2 g
Water	Add 1 L
**Total**	**1 L**

The buffer can be stored at 20°C–25°C for up to 1 year.

**Table:** Phosphate buffer

Reagent	Final concentration	Amount
PBS buffer (×10)	×1	10 mL
NaCl (5 M)	1 M	20 mL
Water	N/A	Add up to 100 mL
**Total**	**N/A**	**100 mL**

The buffer can be stored at 20°C–25°C for up to 1 year.

**Table:** HEPES buffer

Reagent	Final concentration	Amount
HEPES	20 mM	0.477 g
NaCl	150 mM	0.877 g
Water	N/A	Add up to 100 mL
**Total**	**N/A**	**100 mL**

Adjust the pH value to 7.0 at 20°C–25°C with NaOH. The buffer can be stored at 20°C–25°C for up to 1 year.

**Table:** Lipid mixture solution

Reagent	Amount
POPC (1 mM)	25 μL
POPE (1 mM)	24 μL
PI (1 mM)	10 μL
POPS (1 mM)	10 μL
POPA (1 mM)	10 μL
CL (1 mM)	20 μL
Liss Rhod PE (0.1 mM)	10 μL
**Total**	**109 μL**

The solution can be stored in a tightly sealed glass vial at −20°C for up to 6 months.

### IPTG (0.5 M)

Dissolve 1.19 g of IPTG in water up to 10 mL. Divide the sterile filter into 1 mL portions and store at −20°C.

### PMSF (0.1 M)

Dissolve 8.7 mg of PMSF in 500 μL of ethanol just before use. Do not store this solution.

### NaCl (5 M)

Dissolve 292.2 g of NaCl in water up to 1 L. Store at 20°C–25°C for up to 1 year.

### Imidazole (1 M, pH of 8.0)

Dissolve 68.08 g of imidazole in 900 mL of water. Adjust the pH to 8.0 at 20°C–25°C with HCl. Add water up to 1 L and store at 20°C–25°C for up to 1 year.

### Phosphate buffer supplemented with 10 mM maltose

Add 34.2 mg of maltose in 10 mL phosphate buffer. Store at 20°C–25°C for up to 1 month.

### HEPES buffer containing 1 mg/mL bovine serum albumin (BSA)

Dissolve 1.0 mg of BSA in 1 mL of HEPES buffer just before use. Do not store this solution.

### Sample application system

**Figure 1:**
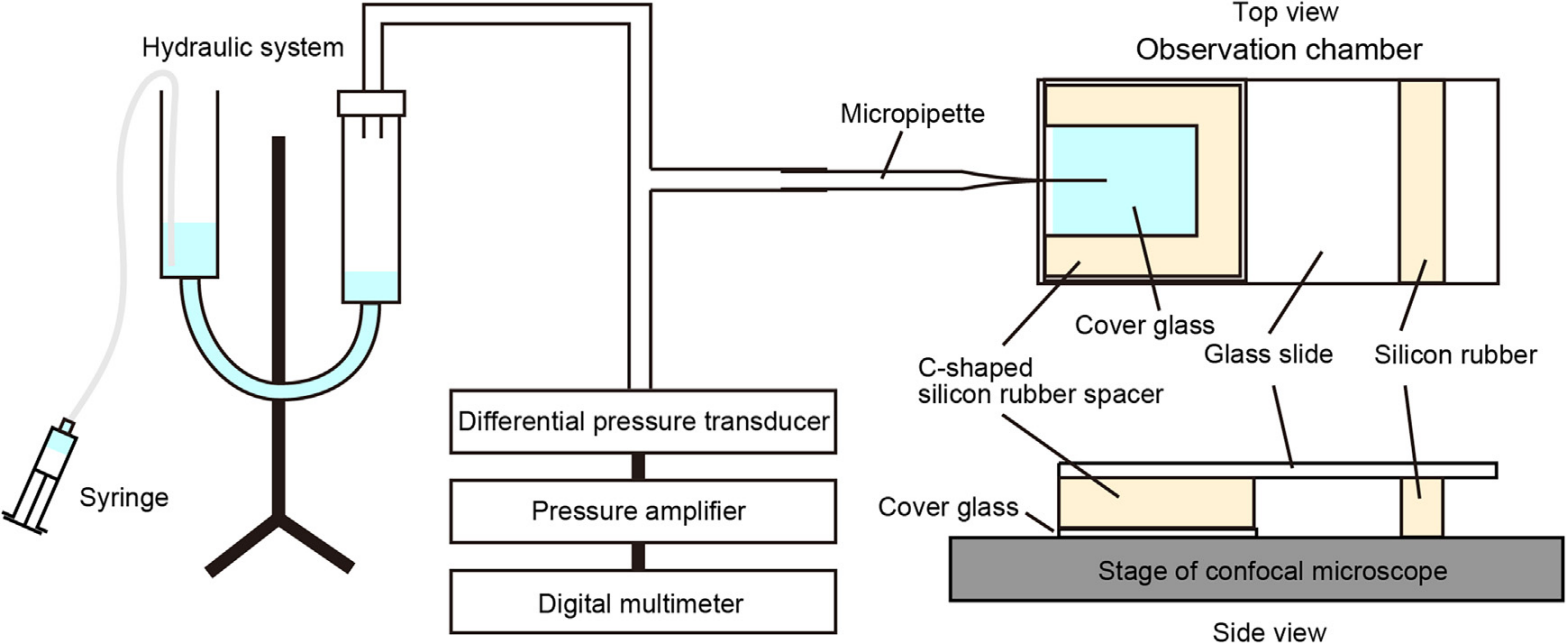
Sample application system

The micropipette is connected to a hydraulic system through a silicone tube to control the sample application via a glass micropipette (Figure 1). The pressure in the system is measured using a differential pressure transducer, a pressure amplifier, and a digital multimeter. The micropipette is attached to a three-dimensional manipulator that is installed on a confocal laser scanning microscope, allowing it to be positioned at a desired location.

## Step-by-step method details

### Excess membrane reservoirs preparation

**Timing: ∼1 h**

Lipid nanotubes are prepared using excess membrane reservoirs consisting of silica beads covered with supported lipid bilayers as previously reported.^3^ The supported lipid bilayers are formed by the adsorption and the fusion of liposomes on the surface of silica beads. The excess membrane reservoirs are thoroughly washed to remove liposomes remaining in the bulk solution.

**Figure 2:**
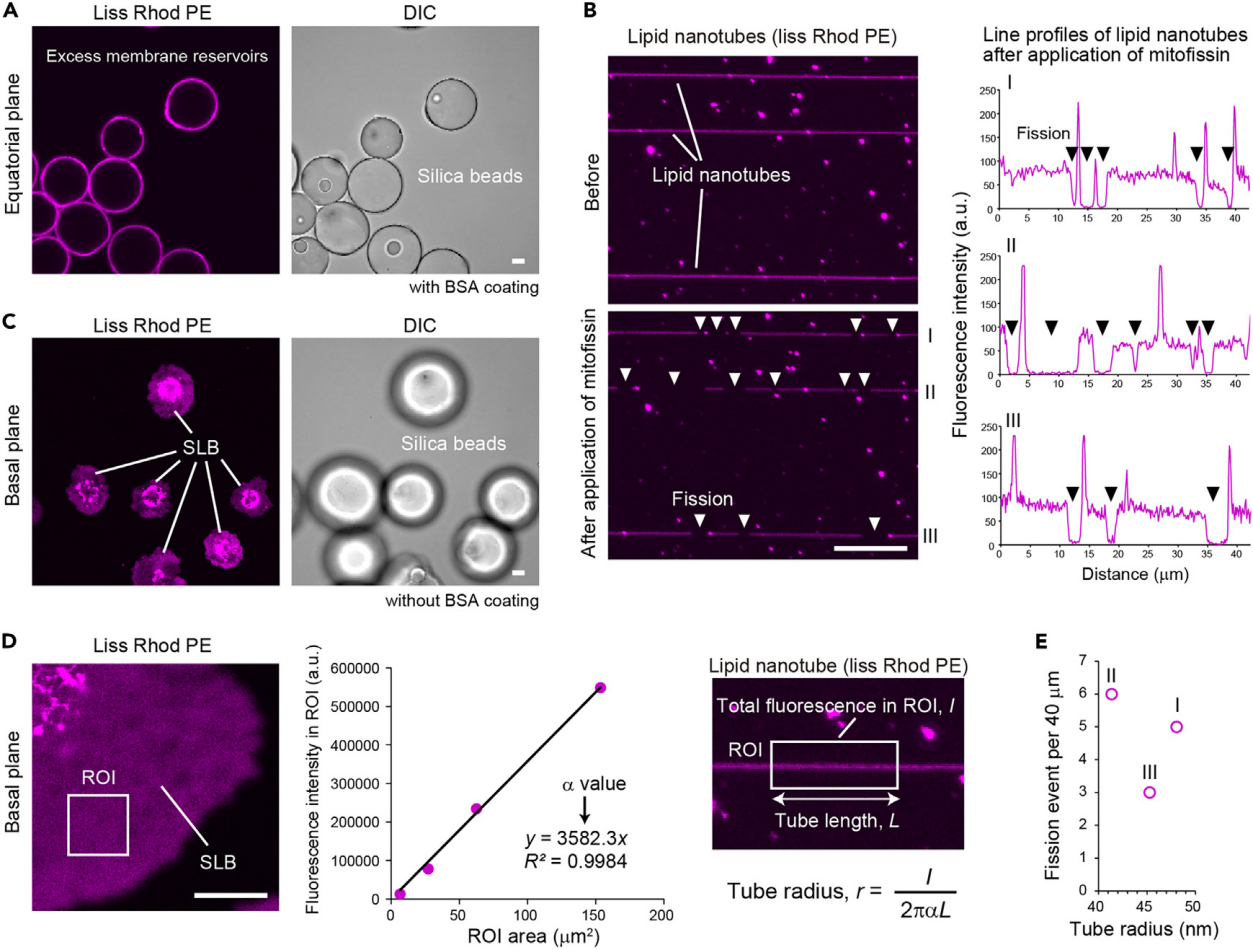
Confocal imaging of membrane fission mediated by mitofissin (A) Excess membrane reservoirs observed in an equatorial plane. (B) Fission of lipid nanotubes by mitofissin. Arrowheads denote parts of fission. (C) Supported lipid bilayers (SLB) from excess membrane reservoirs observed in a basal plane. (D) Estimation of correlation coefficient, α by plotting total fluorescence intensity of ROI against ROI area to quantify tube radius from fluorescence. (E) Tube radius dependency of fission mediated by mitofissin. DIC, differential interference contrast. Scale bars, 10 μm.

1. Prepare excess membrane reservoirs.
  a. Add 40 μL of 5.0 M NaCl to 160 μL of liposome solution containing 250 μM of lipids.
    ***Note:*** Liposome adsorption to the surface of silica beads depends on salt concentration, lipid composition, and the number and size of silica beads.^3^
  b. Add 20 μL of suspension containing silica beads in water to the liposome solution.
    ***Note:*** Solution containing silica beads is recommended to be agitated using a vortex mixer before aspiration.
  c. Agitate the mixture intermittently with a vortex mixer for 30 min at 20°C–25°C.
  d. Add 780 μL of water to the mixture and centrifuge it at 500 × g for 1 min at 25°C.
  e. Remove ∼950 μL of the supernatant, being careful not to aspirate the beads pellet.
  f. Add ∼950 μL of water to the beads pellet and centrifuge it at 500 × g for 1 min at 25°C.
  g. Repeat steps 4e and 4f three times to remove liposomes remaining in the bulk solution.
  h. Remove ∼950 μL of the supernatant, leaving the beads pellet that contains excess membrane reservoirs (Figure 2A).
    **CRITICAL:** Excess membrane reservoirs should be freshly prepared before membrane fission assay to produce lipid nanotubes efficiently.

### Lipid nanotube preparation

**Timing: ∼30 min**

Lipid nanotubes are prepared by rolling excess membrane reservoirs over the bottom of an observation chamber. The rolling can be achieved by simply tilting the observation chamber.

2. Prepare lipid nanotubes.
  a. Prepare an observation chamber assembled with a C-shaped silicone rubber spacer sandwiched between a cover glass and a glass slide (Figure 1).
    ***Note:*** Any observation chamber can be used if the solution does not spill when tilted.
  b. Fill HEPES buffer containing 1 mg/mL of BSA into the observation chamber at 20°C–25°C.
  c. Remove the BSA solution immediately.
    ***Note:*** Longer incubation time will reduce lipid nanotube production likely due to excessive BSA coating on the glass surface.
  d. Fill the observation chamber with HEPES buffer.
  e. Aspirate solution containing excess membrane reservoirs and deposit it at the open side of the observation chamber.
  f. Tilt the observation chamber more than ∼20° for the excess membrane reservoirs to roll slowly toward the inside over the bottom.
  g. Roll the excess membrane reservoirs until they reach the inside wall.
  h. Place the observation chamber horizontally.
  i. Put the observation chamber on the stage of confocal laser scanning microscope to observe lipid nanotubes (Figure 2B).
    **CRITICAL:** Lipid nanotubes must be freshly used within 1 h for membrane fission assay.

### Observation for fission of lipid nanotubes

**Timing: ∼30 min**

Purified mitofissin, SpAtg44, is applied to lipid nanotubes under a confocal laser scanning microscope (Figure 1). The local concentration of SpAtg44 near lipid nanotubes is increased by introducing it via a glass micropipette attached to a three-dimensional manipulator installed on the confocal laser scanning microscope. The sample application is hydraulically controlled while the pressure is monitored using a digital multimeter. The application system is optional to observe membrane fission.

3. Observe membrane fission.
  a. Prepare a glass micropipette with a diameter of ∼20 μm using a puller and a capillary cleaving tool.
  b. Load 100 μM of SpAtg44 in HEPES buffer into the micropipette by aspiration using a dry vacuum pump.
    ***Note:*** Protein concentration required for the observation of membrane fission depends on the protein of interest.
  c. Connect the micropipette to a hydraulic system through a silicone tube.
  d. Set the micropipette on the three-dimensional manipulator.
  e. Adjust the voltage induced by application pressure to be ∼3 V (positive) in a digital multimeter.
    ***Note:*** The value of positive application pressure may depend on the experimental environment.
    ***Note:*** Whether the pressure is positive or negative can be determined by whether the glass micropipette exhales or inhales fluorescently labeled lipid aggregates.
  f. Adjust the micropipette so that the tip is visible under the confocal laser scanning microscope.
  g. Adjust the voltage induced by application pressure to be neutral by reading it using the digital multimeter.
  h. Position the tip of the micropipette near lipid nanotubes.
  i. Apply the additional voltage induced by positive pressure to be up to 300 mV from the neutral value in the digital multimeter to introduce SpAtg44.
  j. Record time-lapse imaging during SpAtg44 application to lipid nanotubes using FV31S-SW software (Figure 2B). Take images of fluorescence from liss Rhod PE excited using a-561 nm laser at every ∼1-s for 15 min with ×60 oil immersion objective lens.
  k. Analyze the time-lapse images using FV31S-SW or Fiji softwares to estimate fission events per tube length (40 μm in our case). Fission sites can be manually counted along the nanotubes based on fluorescence breaks after subtracting background (Figure 2B).
    ***Note:*** The protein of interest can be directly applied using a pipette to lipid nanotubes. Lipid nanotubes are fragile, thus they should be handled with care during the application process to minimize any potential damage.
    ***Note:*** A negative control experiment should be conducted in parallel using a protein like green fluorescent protein irrelevant to fission activity.

### Radius of lipid nanotube estimation

**Timing: ∼1 h**

The radius of a lipid nanotube can be estimated from the fluorescence intensity.^4^ The total fluorescence intensity of a lipid nanotube in the region of interest (ROI) can be described by the equation, I = α(2πrL) where I is the total fluorescence intensity in ROI, α is the correlation coefficient, r is the tube radius, and L is the tube length in ROI. Supported lipid bilayers that have spilled out from an excess membrane reservoir are prepared to estimate the α value. After measuring the total fluorescence intensity and the membrane area of the supported lipid bilayer in ROI, α can be obtained from the equation, I = αA, where A is the membrane area in ROI. Eventually, the fluorescence intensity and the length of the lipid nanotube of interest are measured, thereby calculating the tube radius using α value.

4. Estimate the radius of the lipid nanotube.
  a. Prepare an observation chamber assembled with a C-shaped silicone rubber spacer sandwiched between a cover glass and a glass slide.
    ***Note:*** An observation chamber similar to the one used in membrane fission assay is recommended. The chamber is not coated with BSA.
  b. Fill the observation chamber with HEPES buffer lacking BSA.
  c. Deposit 20 μL of the solution containing excess membrane reservoirs to be scattered in the observation chamber.
    ***Note:*** Excess membrane reservoirs adhere to the glass surface, thereby spilling the membrane in the observation chamber without BSA coating.
  d. Place the observation chamber on the stage of the confocal laser scanning microscope.
  e. Take images of supported lipid bilayers spilled out from excess membrane reservoirs (Figure 2C).
  f. Measure the total fluorescence intensity and the membrane area in ROI after subtracting the background by Fiji software.
  g. Plot the total fluorescence intensity against the membrane area, determining the α value by Excel software (Figure 2D).
  h. Measure the total fluorescence intensity and the length of the lipid nanotube in ROI after subtracting the background by Fiji software. Tube radius, r, is estimated from the equation: r = I/2πLα (Figure 2D).
  i. Plot the fission events per tube length against tube radius to visualize the tube radius dependency of fission activity in the experimental condition (Figure 2E).

## Expected outcomes

This protocol provides the detailed steps for *in vitro* membrane fission observation by yeast mitofissin, SpAtg44. The protocol includes SpAtg44 purification, lipid nanotube preparation using excess membrane reservoirs, and membrane fission observation by SpAtg44. Figure 2 summarizes the expected outcomes. Lots of lipid nanotubes with various radii are produced using excess membrane reservoirs. The lipid nanotubes are severed by SpAtg44 action and are retracted, which is observed in real-time under a confocal laser scanning microscope. The spread of supported lipid bilayers from excess membrane reservoirs in the observation chamber without BSA coating can be used to obtain a correlation coefficient between fluorescence intensity and membrane area in ROI, allowing tube radius estimation.

## Limitations

The protocol enables membrane fission observation by Atg44 using lipid nanotubes as simplified models of biological membranes. However, limitations include the potential inability of lipid nanotubes to fully replicate the intricate lipid organization and membrane curvature of native cellular membranes. Additionally, the absence of other cellular components and regulatory factors in *in vitro* experiments limits the understanding of the complex interplay involved in membrane fission. Therefore, complementing *in vitro* studies with *in vivo* experiments is important to identify the complexity and physiological relevance of membrane fission processes.

## Troubleshooting

### Problem 1

The amount of purified protein is lower than expected (∼10 mg/L culture) (related to Step 1).

### Potential solution

• Check the aeration during culture.
• Check the protein-binding to amylose and Ni-NTA resins by SDS-PAGE.

### Problem 2

Liposome solution is cloudy after sonication (related to Step 2).

### Potential solution

• Check the tuning and the output power of the handheld tip sonicator.
• Check that the temperature is above the phase transition temperature of phospholipids.

### Problem 3

No or weak fluorescence from excess membrane reservoirs (related to Step 3).

### Potential solution

Liposome concentration may be low.

• Increase the concentration of the lipid mixture solution.

The number of silica beads may be excessive.

• Reduce the number of silica beads.

### Problem 4

Lipid nanotubes are not efficiently prepared (related to Step 4).

### Potential solution

BSA coating on the glass surface may be excessive.

• Decrease the BSA concentration.
• Reduce the incubation time for BSA coating.

Rolling of excess membrane reservoir may be fast.

• Reduce the tilt of the observation chamber.

Interaction between the excess membrane reservoir and glass surface may be weak.

• Roll excess membrane reservoirs over a similar path several times.

### Problem 5

No fission is observed (related to Step 5).

### Potential solution

• Check the protein integrity by SDS-PAGE.
• Increase protein concentration.

## Resource availability

### Lead contact

Further information and requests for resources and reagents should be directed to and will be fulfilled by the lead contact, Nobuo N. Noda (nn@igm.hokudai.ac.jp).

### Materials availability

A plasmid used in this paper is available from the lead contact upon request.

### Data and code availability

Any additional information required to reanalyze the data reported in this paper is available from the lead contact upon request.
